# Complete Genome Sequence of *Acidithiobacillus ferrooxidans* YNTRS-40, a Strain of the Ferrous Iron- and Sulfur-Oxidizing Acidophile

**DOI:** 10.3390/microorganisms8010002

**Published:** 2019-12-18

**Authors:** Yu Zhang, Shuang Zhang, Dan Zhao, Yongqing Ni, Weidong Wang, Lei Yan

**Affiliations:** 1 Heilongjiang Provincial Key Laboratory of Environmental Microbiology and Recycling of Argo-Waste in Cold Region, College of Life Science and Biotechnology, Heilongjiang Bayi Agricultural University, Daqing 163319, China; 13163535921@163.com (Y.Z.); zhangshuang@163.com (S.Z.); zhaodan1109@163.com (D.Z.); wwdcyy@sohu.com (W.W.); 2 College of Food Science, Shihezi University, Shihezi 832003, China; nyq_food@shzu.edu.cn

**Keywords:** *Acidithiobacillus ferrooxidans*, complete genome, stress resistance, ferrous iron oxidation, sulfur oxidation

## Abstract

*Acidithiobacillus ferrooxidans* YNTRS-40 (*A. ferrooxidans*) is a chemolithoautotrophic aerobic bacterium isolated from Tengchong hot springs, Yunnan Province, China, with a broad growth pH range of 1.0–4.5. This study reports the genome sequence of this strain and the information of genes related to the adaptation of diverse stresses and the oxidation of ferrous iron and sulfur. Results showed that YNTRS-40 possesses chromosomal DNA (3,209,933-bp) and plasmid DNA (47,104-bp). The complete genome of 3,257,037-bp consists of 3,349 CDS genes comprising 6 rRNAs, 52 tRNAs, and 6 ncRNAs. There are many encoded genes associated with diverse stresses adaptation and ferrous iron and sulfur oxidation such as *rus* operon, *res* operon, *petI*, *petII*, *sqr*, *doxDA*, *cydAB*, and *cyoABCD*. This work will provide essential information for further application of *A. ferrooxidans* YNTRS-40 in industry.

## 1. Introduction

*Acidithiobacillus* usually found in acidic environments with heavy metal and oligotrophic conditions. The members of this genus contribute to the formation of acidic habitats, the acidification of waters [1,2], and the biogeochemical cycle of iron and/or sulfur [3,4]. The *Acidithiobacillus* genus contains eight validated species, including *A. ferrooxidans* [5], *A. ferriphilus* [6], *A. ferrivorans* [7], *A. ferridurans* [8], *A. albertensis* [9], *A. thiooxidans* [10], *A. caldus* [11], and *A. sulfuriphilus* [12]. The former four dominant species can obtain energy for growth by using ferrous iron, elemental sulfur, reduced sulfur compounds, hydrogen, or tetrathionate as electron donors [2,6,8,13,14].

*A. ferrooxidans* has iron- and sulfur-oxidizing abilities and can grow in the environments with high concentrations of metal ions such as pyritic ore bodies, coal deposits and their acidified drainages [15,16,17,18,19,20]. Therefore, it has potential utilization in microbial electrosynthesis systems, eco-friendly bioleaching technology, biological desulfurization and machining of workpieces [21]. Until now, nine *A. ferrooxidans* strain genomes (ATCC 23270, ATCC 53993, Hel18, BY0502, CCM 4253, IO-2C, YQH-1, DLC-5, and RVS1) have been available in the public databases. The genomes of these strains only reported chromosome genomes, while the chromosome genomes and plasmid genomes were not detected at the same time. Additionally, only strains ATCC 23270 and ATCC 53993 were complete genome sequences [20,22,23]. The genome of strain YNTRS-40 reported in this paper is complete and contains a plasmid, which is the first report on the simultaneous acquisition of chromosome genome and plasmid genome. The plasmid genome could be helpful in studying the metabolic characteristics of these microbes.

To further investigate the genetic characteristics and application potential of *A. ferrooxidans* YNTRS-40, its genome was entirely sequenced and annotated. Our findings would be useful for understanding the roles and potential of *A. ferrooxidans* in the field of geobiology, biomedicine, and technology.

## 2. Materials and Methods

### 2.1. Growth Conditions, Genomic DNA Isolation and Morphological Detection

*A. ferrooxidans* YNTRS-40 was isolated from the soil soaked in drainage water in Tengchong hot springs, Yunnan Province, China. Field sampling and geochemical measurements were reported in 2018 [24]. General features of *A. ferrooxidans* YNTRS-40 are shown in additional files in Table S1. This strain was grown in modified 9K medium containing 2.4 g (NH_4_)_2_SO_4_, 0.1 g KCl, 0.5 g K_2_HPO_4_, 0.5 g MgSO_4_·7H_2_O and 0.01 g Ca(NO_3_)_2_, 40 g FeSO_4_·7H_2_O per liter water with pH 1.75, and incubated at 28 °C for 48 h with agitation at 120 rpm on a shaker. Genomic DNA was obtained using sodium dodecyl sulfate (SDS) method and the Blood & Cell Culture DNA midi kit (QIAGEN, Hilden, Germany) following the manufacturer’s standard protocol. Cell morphology of *A. ferrooxidans* YNTRS-40 was detected using scanning electron microscopy (Hitachi S-4800, Ibraraki, Japan).

### 2.2. Genome Sequencing, Assembly and Annotation

The sequencing of the complete genome was performed on Nanopore GridION X5 (Oxford Nanopore Technologies, Oxford, United Kingdom) [25] by constructing a 1D genomic DNA library using the ligation sequencing kit 1D (Oxford Nanopore Technologies, Oxford, United Kingdom). After quality filtering, the high-quality reads were assembled into contigs using the Canu v1.7.11 (Maryland Bioinformatics Labs, Park, MD, USA) [26], and the assembled data was optimized by using Pilon v1.22 (Free Software Foundation, Inc., Boston, MA, USA) [27]. All sequencing project information is in Table S2.

Coding sequences (CDS) in the genome were predicted through Prodigal v2.6.3 (Free Software Foundation, Inc., Boston, Massachusetts) [28]. The tRNAs and rRNAs of the genome were predicted by tRNAscan-SE v2.0 (Lowe Lab, Santa Cruz, CA, USA) [29] and RNAmmer v1.2 (DTU Health Tech, Lyngby, Denmark) [30], respectively. The genome sequence was annotated by the Rapid Annotation Subsystem Technology (RAST) (University of Tennessee, Memphis, TN, USA) [31], the Kyoto Encyclopedia of Genes and Genomes (KEGG) (Kanehisa Laboratories, Kyoto, Japan) [32], the Clusters of Orthologous Groups (COG) (Bethesda Softworks LLC, Rockville, Maryland) [33] and the Gene Ontology (GO) (Stanford University School of Medicine, Stanford, CA, USA) [34]. The circular map of the genome were obtained using Circos v1.7.11(Canada’s Michael Smith Genome Sciences Centre, Vancouver, Canada) [35].

Based on 16S rRNA gene sequence of strain YNTRS-40 (Accession number: MK811409) and other members of *Acidithiobacillus* obtained from GenBank, phylogenetic analysis was carried out by MEGA7 software(Temple University, Philadelphia, PA, USA) [36] using CLUSTAL W (EMBL, Heidelberg, Germany) to perform a multiple alignments [37] and the phylogenetic tree was constructed by the neighbor-joining method [38]. Sequence alignment and ANI analysis were performed using MEGA7 software (Temple University, Philadelphia, PA, USA) [36] and JSpeciesWS (Ribocon GmbH, Bremen, Germany) [39] with default settings.

## 3. Results and Discussion

**Figure 1:**
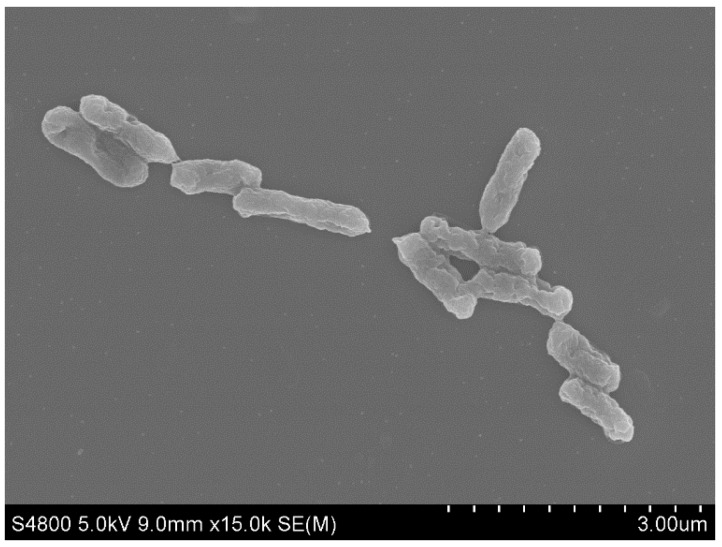
Scanning electron micrograph of *Acidithiobacillus ferrooxidans* YNTRS-40.

**Figure 2:**
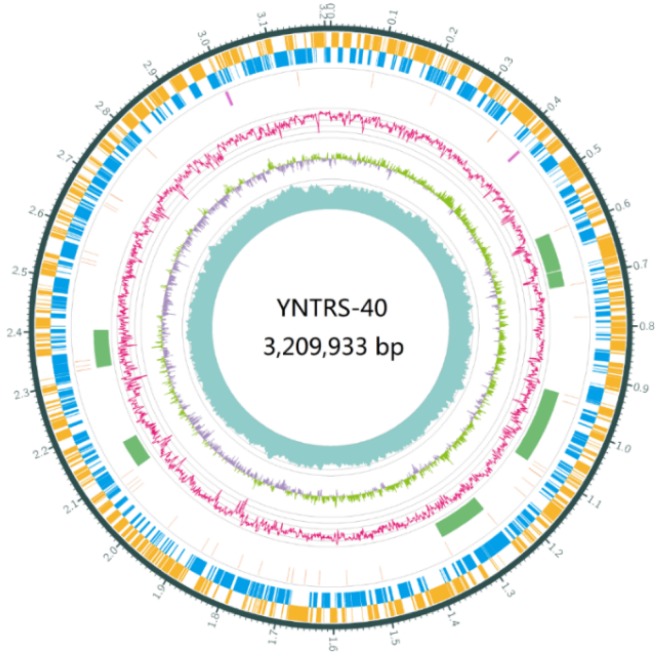
Circular chromosome genome map of *Acidithiobacillus ferrooxidans* YNTRS-40. (From the outside to the center, genes on direct strand, genes on complementary strand, tRNAs (orange), rRNAs (purple), CRISPR (blue), and genomic island (green), GC-skew, sequencing depth are displayed).

*A. ferrooxidans* YNTRS-40 is a Gram-negative non-endospore-forming chemolithoautotrophic aerobic bacteria in the order *Acidithiobacillales* of the class *Acidithiobacillia* (Table S1). It possesses resistance against heavy metal and oligotrophic conditions. Although several *Acidithiobacillus* species with the capacity of iron and sulfur oxidization were identified from acidic environments, their genetic features associated with the resistance to the extreme environment were ambiguous [4]. In this study, *A. ferrooxidans* YNTRS-40 was isolated and its genome was sequenced to analyze the stress resistance. Microscopically, YNTRS-40 cells displayed rod-shaped and were 0.28–0.40 μm in width and 1.00–1.68 μm in length (Figure 1). This strain grew to a logarithmic stage fastly after 48 h under aerobic conditions at pH 1.75, 28 °C and 120 rpm in a shaker with a modified 9K medium. The complete genome sequences have been submitted to GenBank under the accession number CP040511 (chromosome; Figure 2) and CP040512 (plasmid).

The genome size of *A. ferrooxidans* YNTRS-40 is 3,257,037-bp, and the genome contains one circular chromosome of 3,209,933-bp with 58.54% GC content and one circular plasmid (47,104-bp with 56.43% GC content). The circular chromosome comprised 3349 predicted CDS genes, 6 rRNAs, 52 tRNAs and 6 ncRNAs (Table 1), and the circular plasmid contained 70 predicted CDS genes. The statistics and properties of the genome are summarized in Table 1. Total 2015 genes identified from the chromosome were classified into 26 functional categories based on the Cluster of Orthologous Groups (COG; Table 2) [40]. Among all categories, the inorganic ion transport and metabolism category (P, 6.70%), the energy production and conversion category (C, 6.40%) and the defense mechanisms category (V, 4.47%) indicate that the strain YNTRS-40 can grow in the environment with high concentrations of metal ion.

**Figure 3:**
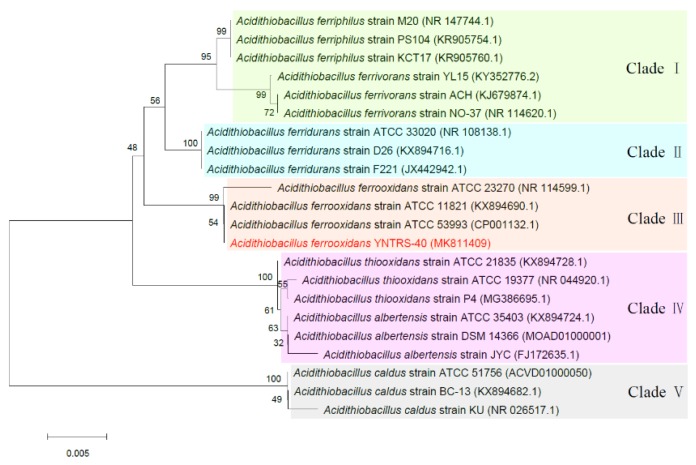
Phylogenetic tree based on the 16S rRNA gene sequence of *Acidithiobacillus ferrooxidans* YNTRS-40 and its relatives. Bootstrap values were calculated by MEGA7 using the neighbor-joining method from 1000 replications. Bar, 0.005 nucleotide substitutions per nucleotide position.

Based on 16S rRNA gene sequence analysis, it can be seen that all strains clustered separately into different clades, such as *A. ferriphilus* (Clade I), *A. ferrivorans* (Clade I), *A. ferridurans* (Clade II), *A. ferrooxidans* (Clade III), *A. thiooxidans* (Clade IV), *A. albertensis* (Clade IV), and *A. caldus* (Clade V). This finding was similar to a study by Zhang et al. [41] and slightly different from literature, in which *A. ferridurans*, *A. thiooxidans,* and *A. albertensis* clustered into the common clade [2]. The strain YNTRS-40 appeared to represent a coherent group with *Acidithiobacillus ferrooxidans* ATCC 11821 and *Acidithiobacillus ferrooxidans* ATCC 53993 (Figure 3). The 16S rRNA gene sequence similarities between the strain YNTRS-40 and the closest relative and the result of ANI analysis among *A. ferrooxidans* strains, including the strain YNTRS-40, are shown in Tables S3 and S4.

### 3.1. Genomic Features Related to Adaptation to Diverse Stresses

*Acidithiobacillus* spp. possess extreme environmental resistance, and they can adjust their survival, colonization, growth, and development to extremely acidic conditions (grow optimally at pH 2.0 and survive in pH 1.0–4.5) [42,43,44]. To balance the extracellular and intracellular environment heterogeneity in extremely acidic habitats containing heavy metal ions, these acidophilic microorganisms diverge and evolve to possess the acid and metal resistance [40].

Genome analysis using the Rpsblast and the Interproscan v5.30–69.0 [45] revealed many functional genes involved in the adaptation of strain YNTRS-40 to extreme environments (Table 2 and Tables S5 and S6). Among them, the Cus systems are critical in copper resistance [46]. The expression of proteins such as oxidoreductase and transferase in the cell should be regulated under the environment containing sulfur and metal ions. It has been documented that the oxidoreductase and transferase not only participate in energy generation but also enhance tolerance to environmental stress [47,48,49]. Based on the category of biological process in Table S5, the gene function of strain YNTRS-40, such as response to extracellular stimulus, cellular response to stress, and response to oxidative stress, indicated that it could cope with extreme environmental stress.

Based on the COG analysis (Table 2), the functional genes related to defense mechanisms (V), cell wall/membrane/envelope biogenesis (M), amino acid transport and metabolism (E), inorganic ion transport and metabolism (P) and general function prediction only (R) were slightly more than the other genes. These revealed that this strain exhibited excellent environmental adaptability and has potential applications in the ecological industry, such as sulfur removal from gases, metal extraction from electronic waste [21]. Additionally, the genes associated with function unknown (S) indicated that strain YNTRS-40 might possess some new genes [50].

The plasmid usually contains the genes related to secondary metabolism according to the characteristics of microorganisms [51]. There were 70 CDSs in the circular plasmid of strain YNTRS-40, and 39 CDSs of them were predicted as hypothetical proteins, and the rest were found to be involved in metabolism and defense (Table S7). The RAST annotation results showed that the plasmid comprised all kinds of secondary metabolism-related genes, transcriptional regulatory genes, transposase-related genes, mobile element protein-related genes, and stress-tolerance genes. These indicated that the primary metabolism-related genes were not present in this plasmid, and the presence of plasmid might favor the adaptation of this strain to environmental stress.

### 3.2. Genomic Features Related to the Oxidation of Ferrous Iron and Sulfur

*A. ferrooxidans* can gain energy from the oxidation of Fe^2+^ for growth and survival [5,20]. During the oxidation of Fe^2+^, most of the electrons are transferred to O_2_ along the potential gradient, which called downhill potential gradient, while a small part of electrons is transmitted conversely along the potential gradient, which named uphill potential gradient [52]. In the latter process, the NAD(P)H is generated and involved in CO_2_ fixation and aerobic metabolism [20,52,53]. These two electron transfer pathways, namely the downhill and the uphill potential gradients, are interrelated [52].

The KEGG analysis showed that several coding genes related to the downhill and the uphill electron transfer pathway existed in the strain YNTRS-40 (Table S8). Among them, the *rus* operon, which consists of *cyc2*, *cyc1*, *cup*, *coxB*, *coxA*, *coxC*, *coxD*, and *rus* genes, were found to be involved in electron transfer in the downhill electron pathway [54]. The previous study suggested that the expression and regulation of the *rus* operon are associated with the substrate electron donor in the environment [54]. The operon might be activated persistently when bacteria use Fe^2+^ as an electron donor, but only expressed transitorily during the period of early logarithmic growth when cells take S as an electron donor [55,56]. Additionally, the *pet I*, *pet II* and *res* operon were found in the genome of the strain YNTRS-40. Among them, *pet I* encodes a bc1 complex that participates in the inverse electron transfer when the strain uses Fe^2+^ as a substrate, and *pet II* encodes another bc1 complex, which is responsible for forwarding electron transfer when S is used as substrate [57]. The *res* operon near the *pet* operon encodes ResB and ResC protein, which might serve as a molecular chaperone in the maturation process of the *c1* cytochrome of the *bc1* complex [58].

*A. ferrooxidans* also can obtain the energy required for growth by oxidizing reduced sulfur. Sulfur was found to be a more favorable energy source than Fe^2+^ because it can provide more ATP than Fe^2+^ at the same molar level [59,60]. The KEGG results indicated that some genes involved in sulfur oxidation, including *sqr*, *doxDA*, *cydAB*, and *cyoABCD* genes, which code sulfide quinone reductase, thiosulfate quinone oxidoreductase and thiosulfate dehydrogenase, respectively (Table S8). These genes might be upregulated in strain YNTRS-40 when sulfur is used as a substrate. Additionally, the enzymes encoded by these genes were coupled to a respiratory chain and occurred at different nodes in the respiratory chain [61]. These results suggested that the strain YNTRS-40 has the potential for industrial application through iron and sulfur-oxidizing such as metal bioleaching, gas desulfurization, and bioremediation.

## 4. Conclusions

The genome of *A. ferrooxidans* YNTRS-40 revealed that the strain could grow well under extremely acidic conditions containing heavy metal ions and has the ability to remove sulfur from gases and extract metal from solids since it contained various genes participating in the adaptation to environmental stress and the oxidation of ferrous iron and sulfur. This paper is a simultaneous first in reporting the chromosome genome and the plasmid genome of *A. ferrooxidans*. It could be helpful to research the metabolic characteristics and commercial application potential of *A. ferrooxidans* YNTRS-40 in the future.

## Figures and Tables

**Table 1:** Genome statistics of *Acidithiobacillus ferrooxidans* YNTRS-40.

Attribute	Value	% of Total ^1^
Genome size (bp)	3,257,037	100.00
DNA coding (bp)	2,940,490	90.28
DNA G + C (bp)	1,905,651	58.51
DNA scaffolds	2	100.00
Total genes	3419	100.00
Protein coding genes	3349	97.95
RNA genes	70	2.05
Pseudo genes	NA ^2^	NA ^2^
Genes in internal clusters	8	16.21
Genes with function prediction	1692	50.52
Genes assigned to COGs	1793	53.54
Genes with Pfam domains	2539	75.81
Genes with signal peptides	NA ^2^	NA ^2^
Genes with transmembrane helices	NA ^2^	NA ^2^
CRISPR repeats	0	0

^1^ The total is based on either the size of the genome in base pairs or the total number of protein coding genes in the annotated genome; ^2^ NA, not applicable.

**Table 2:** Number of genes associated with general COGs functional categories.

Code	Value	% Age ^1^	Description
J	172	5.14	Translation, ribosomal structure, and biogenesis
A	0	0	RNA processing and modification
K	118	3.52	Transcription
L	126	3.76	Replication, recombination, and repair
B	1	0.03	Chromatin structure and dynamics
D	29	0.87	Cell cycle control, Cell division, chromosome partitioning
Y	0	0	Nuclear structure
V	90	2.69	Defense mechanisms
T	87	2.60	Signal transduction mechanisms
M	162	4.84	Cell wall/membrane/envelope biogenesis
N	20	0.60	Cell motility
Z	0	0	Cytoskeleton
W	14	0.42	Extracellular structures
U	62	1.85	Intracellular trafficking, secretion, and vesicular transport
O	96	2.87	Posttranslational modification, protein turnover, chaperones
C	129	3.85	Energy production and conversion
G	93	2.78	Carbohydrate transport and metabolism
E	137	4.09	Amino acid transport and metabolism
F	53	1.58	Nucleotide transport and metabolism
H	108	3.22	Coenzyme transport and metabolism
I	72	2.15	Lipid transport and metabolism
P	135	4.03	Inorganic ion transport and metabolism
Q	34	1.02	Secondary metabolites biosynthesis, transport, and catabolism
R	138	4.12	General function prediction only
S	51	1.52	Function unknown
X	88	2.63	Mobilome: prophage, transposons
-	1334	39.83	Not in COGs

^1^ The total is based on the total number of protein coding genes (3349) in the genome.

## Supplementary Materials

https://www.mdpi.com/2076-2607/8/1/2/s1. Table S1: Classification and general features of *Acidithiobacillus ferrooxidans* YNTRS-40 according to the MIGS recommendations, Table S2: Genome sequencing project information for *Acidithiobacillus ferrooxidans* YNTRS-40, Table S3: The 16S rRNA gene sequence similarities between the strain YNTRS-40 and the closest relative, Table S4: The result of ANI analysis among *Acidithiobacillus ferrooxidans* strains including the strain YNTRS-40, Table S5: Partial GO annotation of coding proteins in *Acidithiobacillus ferrooxidans* YNTRS-40 chromosome genome, Table S6: Identified metal resistance genes in Acidithiobacillus ferrooxidans YNTRS-40, Table S7: RAST categories of CDSs in *Acidithiobacillus ferrooxidans* YNTRS-40 plasmid genome, Table S8: Identified genes associated with ferrous iron and sulfur oxidation in *Acidithiobacillus ferrooxidans* YNTRS-40.
