# Supplementary material for: Complete Genome Sequence of Acidithiobacillus ferrooxidans YNTRS-40, a Strain of the Ferrous Iron- and Sulfur-Oxidizing Acidophile
Source: Microorganisms. 2019 Dec 18;8(1):2. doi: 10.3390/microorganisms8010002 (PMC7023503; doi:10.3390/microorganisms8010002)
Supplement: Supplementary file 1 [file microorganisms-08-00002-s001.zip › microorganisms-660572-supplementary/Supplementary information.docx]

**Table S1.** Classification and general features of *Acidithiobacillus ferrooxidans* YNTRS-40 according to the MIGS recommendations [1].

| **MIGS ID** | **Property** | **Term** | **Evidence code^a^** |
| --- | --- | --- | --- |
|  | Classification | Domain *Bacteria* | TAS [2] |
|  |  | Phylum *Proteobacteria* | TAS [3] |
|  |  | Class *Acidithiobacillia* | TAS [3] |
|  |  | Order *Acidithiobacillales* | TAS [3] |
|  |  | Family *Acidithiobacillaceae* | TAS [3] |
|  |  | Genus *Acidithiobacillus* | TAS [4] |
|  |  | Species *Acidithiobacillus ferrooxidans* | TAS [4] |
|  |  | Strain YNTRS-40 | IDA |
|  | Gram stain | Negative | IDA |
|  | Cell shape | Rod-shaped | TAS [4] |
|  | Motility | Motile | IDA |
|  | Sporulation | Non-sporulating | NAS |
|  | Temperature range | 10 to 47^o^C | NAS |
|  | Optimum temperature | 30^o^C | NAS |
|  | pH range; Optimum | 1.0-4.5; 2.0 | NAS |
|  | Carbon source | Autotroph | NAS |
| MIGS-6 | Habitat | Soil | TAS [4] |
| MIGS-6.3 | Salinity | Not reported | IDA |
| MIGS-22 | Oxygen requirement | Aerobic | NAS |
| MIGS-15 | Biotic relationship | Free-living | NAS |
| MIGS-14 | Pathogenicity | Non-pathogen | NAS |
| MIGS-4 | Geographic location | China: Yunnan | NAS |
| MIGS-5 | Sample collection | Not reported | IDA |
| MIGS-4.1 | Latitude | 25° 01′ 00′′ N | NAS |
| MIGS-4.2 | Longitude | 98° 51′ 00′′ E | NAS |
| MIGS-4.4 | Altitude | Not reported | IDA |

^a^ Evidence codes-IDA: Inferred from Direct Assay, TAS: Traceable Author Statement (i.e., a direct report exists in the literature), NAS: Non-traceable Author Statement (i.e., not directly observed for the living, isolated sample, but based on a generally accepted property for the species, or anecdotal evidence). These evidence codes are from the Gene Ontology project [5].

**Table S2.** Genome sequencing project information for *Acidithiobacillus ferrooxidans* YNTRS-40.

| **MIGS ID** | **Property** | **Term** |
| --- | --- | --- |
| MIGS-31 | Finishing quality | Finished |
| MIGS-28 | Libraries used | 1D Genomic |
| MIGS-29 | Sequencing platforms | Oxford Nanopore GridION |
| MIGS-31.2 | Fold coverage | 152.25× |
| MIGS-30 | Assemblers | CANU 1.7.11 |
| MIGS-32 | Gene calling method | Pilon 1.22 |
|  | Genbank ID | CP040511, CP040512 |
|  | GenBank Date of Release | 2020-06-11 |
|  | BIOPROJECT | PRJNA543563 |
| MIGS-13 | Project relevance | Industrial |
|  | Source Material Identifier | *Acidithiobacillus ferrooxidans* YNTRS-40 |

**Table S3.** Partial GO annotation of coding proteins in *Acidithiobacillus ferrooxidans* YNTRS-40 chromosome genome.

| **Function (level 1)** | **Function (level 4)** | **Value** |
| --- | --- | --- |
| Molecular function | Oxidoreductase activity, acting on a sulfur group of donors | 14 |
|  | Oxidoreductase activity, acting on hydrogen as donor | 2 |
|  | Oxidoreductase activity, oxidizing metal ions | 2 |
|  | Oxidoreductase activity, acting on iron-sulfur proteins as donors | 5 |
|  | Transferase activity, transferring sulfur-containing groups | 7 |
|  | Ferrochelatase activity | 1 |
|  | Sirohydrochlorin cobaltochelatase activity | 1 |
|  | Intramolecular oxidoreductase activity | 8 |
|  | Ligase activity, forming carbon-sulfur bonds | 5 |
|  | Sulfur compound transmembrane transporter activity | 3 |
|  | Iron-sulfur cluster binding | 47 |
| Cellular component | Oxidoreductase complex | 11 |
| Biological process | Sulfur compound metabolic process | 31 |
|  | Sulfate reduction | 1 |
|  | Regulation of cellular response to stress | 2 |
|  | Cellular response to extracellular stimulus | 5 |
|  | Cellular response to stress | 52 |
|  | Secondary metabolic process | 3 |
|  | Oxidation-reduction process | 171 |
|  | Regulation of response to stress | 2 |
|  | Defense response | 10 |
|  | Response to oxidative stress | 3 |
|  | Response to heat | 2 |
|  | Response to water deprivation | 1 |
|  | Response to starvation | 2 |
|  | Response to temperature stimulus | 2 |
|  | Response to acid chemical | 1 |
|  | Response to arsenic-containing substance | 2 |
|  | Response to transition metal nanoparticle | 7 |
|  | Detoxification of mercury ion | 3 |

**Table S4.** RAST categories of CDSs in *Acidithiobacillus ferrooxidans* YNTRS-40 plasmid genome.

| **Function** | **Number** |
| --- | --- |
| Hypothetical protein | 39 |
| Programmed cell death antitoxin YdcD | 1 |
| Programmed cell death toxin YdcE | 1 |
| Transposase | 2 |
| Transcriptional regulator, LysR family | 1 |
| Multidrug efflux system EmrAB-OMF, inner-membrane proton/drug antiporter EmrB (MFS type) | 1 |
| Outer membrane factor (OMF) lipoprotein associated wth EmrAB-OMF efflux system | 1 |
| Multidrug efflux system EmrAB-OMF, membrane fusion component EmrA | 1 |
| Putative bacterial haemoglobin  Flavohemoprotein  Repair of Iron Centers di-iron protein  Glycine cleavage system H protein like protein HTH_1877  Heterodisulfide reductase subunit B like protein HTH_1882  Motif=eukaryotic putative RNA-binding region RNP-1 signature  4Fe-4S dicluster domain / Domain of unknown function DUF3470  Transketolase (EC 2.2.1.1)  Transcriptional regulator, Crp/Fnr family  Hydroxylamine reductase (EC 1.7.99.1)  Oxidoreductase FAD/NAD(P)-binding domain protein  NnrS protein involved in response to NO  Transposase, wcw_0483 family  Phage DNA invertase  Mobile element protein  VapB protein (antitoxin to VapC)  VapC toxin protein  NERD domain protein  Retron-type RNA-directed DNA polymerase (EC 2.7.7.49) | 1  1  1  1  1  1  1  1  1  1  1  2  1  1  3  2  1  1  1 |

**References:**

1. Field D, Garrity G, Gray T, Morrison N, Selengut J, Sterk P, et al. The minimum information about a genome sequence (MIGS) specification. Nature Biotechnology. 2008;26(5):541-7.

2. Woese CR, Kandler O, Wheelis ML. Towards a natural system of organisms: proposal for the domains *Archaea*, *Bacteria*, and *Eucarya*. Proceedings of the National Academy of Sciences. 1990;87(12):4576-9.

3. Garrity G, Staley JT, Boone DR, De Vos P, Goodfellow M, Rainey FA, Garrity GM, Schleifer K-H. *Proteobacteria* phyl. nov. In: Brenner DJ, Krieg NR, Staley JT, Garrity GM, editors. Bergey's Manual of Systematic Bacteriology. New York: Springer; 2005.

4. Xian Z, Xueduan L, Liangzhi L, Guanyun W, Danli Z, Yili L, Bo M. Phylogeny, divergent evolution, and speciation of sulfur-oxidizing *Acidithiobacillus* populations. BMC Genomics. 2019;20(1):438.

5. Ashburner M, Ball CA, Blake JA, Botstein D, Butler H, Cherry JM, et al. Gene Ontology: tool for the unification of biology. Nature Genetics. 2000;25(1):25-9.
